# MFDA-YOLO: A multiscale feature fusion and dynamic alignment network for UAV small objects detection

**DOI:** 10.1371/journal.pone.0337810

**Published:** 2025-12-05

**Authors:** Dan Tian, Xiao Wang, Dongxin Liu, Ying Hao

**Affiliations:** School of Intelligent Science and Information Engineering, Shenyang University, Shenyang, Liaoning Province, China; Leibniz University Hannover, GERMANY

## Abstract

Standard detectors such as YOLOv8 face significant challenges when applied to aerial drone imagery, including extreme scale variations, minute targets, and complex backgrounds. Their generic feature fusion architecture is prone to generating false positives and missing small objects. To address these limitations, we propose an improved MFDA-YOLO model based on YOLOv8. The model introduces an Attention-based Intra-scale Feature Interaction (AIFI) module in the backbone network to enhance high-level feature interactions, improve the adaptation to multi-scale targets, and strengthen feature representation. In the neck network, we design the Drone Image Detection Pyramid (DIDP) network, which integrates a space-to-depth convolution module to efficiently propagate multi-scale features from shallow to deep layers. By introducing an omni-kernel module in the cross-stage partial network for image recovery, DIDP can enhance global contextual awareness and eliminate the computational burden to extend the traditional P2 detection layer. Aiming at the problem of insufficient synergy between localization and classification tasks in the detection head, we design the Dynamic Alignment Detection Head (DADH). DADH can achieve cross-task representation optimization through multi-scale feature interaction learning and a dynamic feature selection mechanism, which significantly reduces model complexity and maintains detection accuracy. In addition, we employ the WIoUv3 loss function to dynamically adjust the focusing coefficients and enhance the model’s ability to distinguish small targets. Extensive experimental results demonstrate that MFDA-YOLO outperforms existing state-of-the-art methods such as YOLOv11 and YOLOv13 across the VisDrone2019, HIT-UAV, and NWPU VHR-10 datasets. Particularly on the VisDrone2019 dataset, MFDA-YOLO surpasses the baseline YOLOv8n model, achieving a 4.4 percentage point improvement in mAP0.5 and a 2.7 percentage point increase in mAP0.5:0.95. Furthermore, it reduces parameters by 17.2%, effectively lowering both false negative and false positive rates.

## 1. Introduction

With the rapid development of science and technology, Unmanned Aerial Vehicles (UAVs) have been widely used in the fields of agriculture, disaster relief and transport due to their flexibility, low cost and ease of operation [1]. However, UAV object detection often faces challenges such as scale variations, dynamic viewpoints, complex backgrounds, and dense target overlaps, which make traditional detection frameworks less effective. Therefore, the development of a lightweight and high-precision algorithm for UAV small target detection in complex environments has great research value and application potential [2].

The accuracy and efficiency of object detection algorithms have been significantly enhanced by the broad application of deep learning techniques, particularly Convolutional Neural Networks, surpassing traditional methods [3]. Deep learning-based object detection algorithms generally fall into two categories: one-stage algorithms (e.g., You Only Look Once (YOLO)) and two-stage algorithms (e.g., the R-CNN series) [4].

The one-stage object detection algorithm predicts the target location and category directly on the original image through an end-to-end regression strategy, which avoids the computational overhead of generating candidate regions. This focus on speed, however, reveals inherent limitations when detecting small, occluded objects in aerial imagery. Redmon et al. [5] proposed the YOLO algorithm, which frequently fails to detect the small-sized targets common in aerial drone perspectives. This failure stems from its inherent limitations in feature extraction and poor adaptability to scale variations. Other methods also struggle in densely populated aerial scenes. For instance, Law and Deng [6] proposed the keypoint-based CornerNet, and Tian et al. [7] proposed the anchor-free FCOS detector, but both approaches perform poorly. Severe occlusions and overlapping center points disrupt precise localization, while the anchor-free design may cause mismatches between predicted bounding boxes and actual object dimensions. Tan et al. [8] proposed EfficientDet, which attempts to enhance performance through more complex feature fusion networks. However, its high computational cost makes real-time deployment on resource-constrained UAV platforms challenging. Similarly, Zhang et al. [9] proposed YOLO-MFD, which introduces multi-dimensional attention weighting in the detection head to enhance feature focus. However, this approach brings significant computational overhead, and its dynamic spatial alignment capability remains insufficient for extremely small aerial objects.

To overcome the precision limitations inherent in one-stage detectors, researchers have naturally explored high-precision two-stage algorithms. However, such methods commonly suffer from excessive computational overhead, which conflicts with the real-time inference demands of drone terminals. For instance, Cai et al. [10] proposed Cascaded R-CNN, a method that optimizes detection box positioning accuracy by employing a multi-stage mechanism that progressively increases the IoU threshold. However, it is precisely this cascade process that results in its substantial computational cost. At the level of feature representation enhancement, He et al. [11] proposed the Feature Pyramid Network (FPN), which leverages cross-level fusion to improve multi-scale feature characterization. However, extremely small objects in UAV imagery suffer severe feature decay as they propagate through deep networks. This results in a loss of semantic information, which FPN struggle to effectively compensate for. Furthermore, the problem persists even when employing advanced backbone architectures. While Liu et al. [12] proposed the Swin Transformer, which effectively models global contextual information through a hierarchical sliding window attention mechanism. However, its fixed window segmentation struggles to effectively identify the multi-scale, irregular micro-objects commonly found in drone imagery, posing a risk of missed detections.

In summary, developing algorithms for UAV detection that balance accuracy, efficiency, and lightweight design remains a core challenge. Because UAVs have real-time requirements, more efficient one-stage detectors are a more promising research direction [13]. Therefore, this study chooses the YOLOv8 [14] algorithm as the baseline for its excellent balance between speed and accuracy. Despite this strength, it still struggles with the small targets and complex backgrounds common in drone detection, reflecting the inherent limitations of one-stage detectors. To address this issue, we propose MFDA-YOLO, aiming to significantly enhance the model’s multi-scale feature capabilities while strictly controlling computational complexity. The main contributions of this study are as follows:

(1) Detection of small, densely packed targets in drone aerial photography relies on precise spatial details for accuracy. These details are precisely the elements that the Spatial Pyramid Pooling Fast (SPPF) module tends to blur, leading to missed detections. To address this, we utilize the Attention-based Intra-scale Feature Interaction (AIFI) module to replace the SPPF module in the backbone network. The AIFI module captures dependencies between same-scale features using a self-attention mechanism, which enhances the focusing ability of the network.
(2) Small object detection for drones relies on P2 layer details, but this incurs high computational costs. To address this issue, we propose the Drone Image Detection Pyramid (DIDP). The model employs SPD-Conv to perform lossless downsampling on the P2 layer, reorganizing spatial structural information into the channel dimension. Additionally, we design the C-OKM module to recover missed image details, which provides richer features for subsequent feature fusion.
(3) To further mitigate the issue of excessive parameter complexity introduced by the P2 detection layer, we propose the Dynamic Alignment Detection Head (DADH). This module first employs shared convolutions for feature extraction, thereby maximizing control over the model’s parameter count. Subsequently, the task decomposition is used to extract corresponding features for each task. By integrating deformable convolutions with a dynamic weight selection mechanism, adaptive feature processing was achieved, effectively mitigating conflicts between tasks.
(4) Given the widespread issue of lightweight detectors struggling to converge when confronted with large volumes of low-quality samples, we have replaced the baseline CIOU loss function with the WIoUv3 loss function. It employs dynamic coefficients to direct the model’s attention to hard-to-distinguish small targets. The WIoUv3 effectively mitigates oscillations through adaptive normalization.

## 2. Technical background

**Fig 1:**
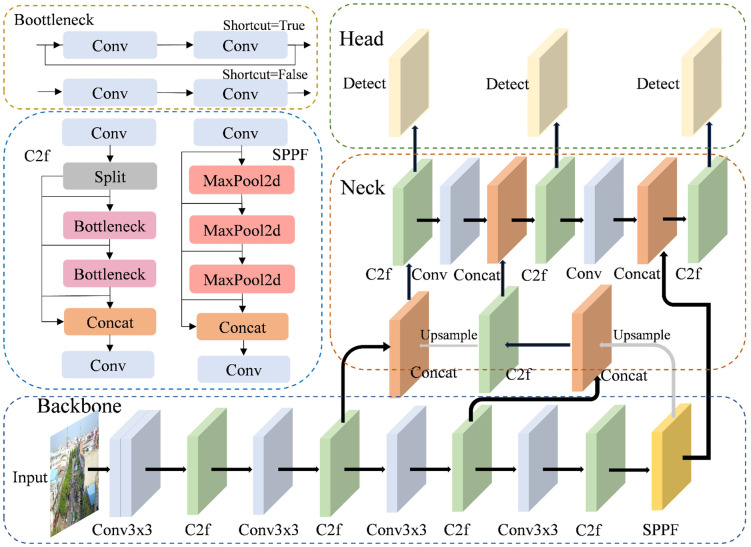
Structure of the YOLOv8 model.

This section presents a comprehensive analysis of YOLOv8’s network architecture and explains the functionality of its component modules. Building upon this foundation, it examines the inherent limitations encountered when applying the model to specific tasks. Compared to previous YOLO models, YOLOv8 has refined and optimized its network structure. As shown in Fig 1, its core architecture includes three modules: Backbone, Neck and Head.

### 2.1 Backbone

The backbone network comprises convolutional layers, C2f layers, and an SPPF, which form the core of its feature extraction design. The backbone network extracts multi-level feature information from the input image by working in concert with multiple convolutional, pooling and activation function layers. This process achieves deep feature extraction and gradually reduces the size of the feature map, which ultimately provides rich semantic support for the subsequent detection head [15].

The C2f layer improves the efficiency of feature expression and detection accuracy through multi-scale feature fusion and adaptive size adjustment mechanisms. The mechanism generates feature maps by alternating 1 × 1 and 3 × 3 convolutions and integrates them through gradient shunting connections to enhance the information flow and keep the network lightweight.

The SPPF module replaces the Spatial Pyramid Pooling module used in earlier versions of YOLO. Unlike the multi-scale pooling kernels of the Spatial Pyramid Pooling module, SPPF processes the feature maps by applying small pooling kernels sequentially [16]. This cascading structure significantly improves computational efficiency and maintains the original sensory field. Subsequently, the SPPF module concatenates the original input features with multi-stage pooled outputs along the channel dimension to generate fixed-dimensional feature vectors. These feature vectors are fed directly into the downstream network for feature extraction.

### 2.2 Neck

The neck network uses the C2f module in conjunction with the path aggregation network and feature pyramid network. Its core function is to analyze and fuse features from the backbone network, which boosts the model’s ability to detect targets of varying sizes [17]. Moreover, the neck network performs multi-scale fusion of the feature maps through the path aggregation network and C2f modules to efficiently aggregate shallow information into deep features.

### 2.3 Head

YOLOv8 implements a decoupled-head design, where classification and regression tasks are processed through two distinct specialized branches. The classification branch processes category-specific features through 1 × 1 convolutional layers for object recognition. The regression branch extracts spatial coordinates and scales via dedicated convolutional operations for object location.

### 2.4 Limitations analysis

Despite YOLOv8’s strong performance on general detection tasks, its standard architecture exhibits inherent limitations when applied to the unique challenges of drone-based object detection. A primary issue stems from the backbone and neck, where continuous downsampling operations, intended to enlarge receptive fields, compromise the high-resolution spatial details essential for localizing these small targets from a distance.

Furthermore, the head’s fixed receptive fields fail to adequately handle the drastic scale variations typical of drone footage. Consequently, a critical misalignment emerges between the semantic features for classification and the precise spatial cues for regression, leading to significant performance degradation on challenging small targets viewed from an aerial perspective. This work is dedicated to addressing these specific architectural deficiencies for robust drone-based detection.

## 3. Methods

**Fig 2:**
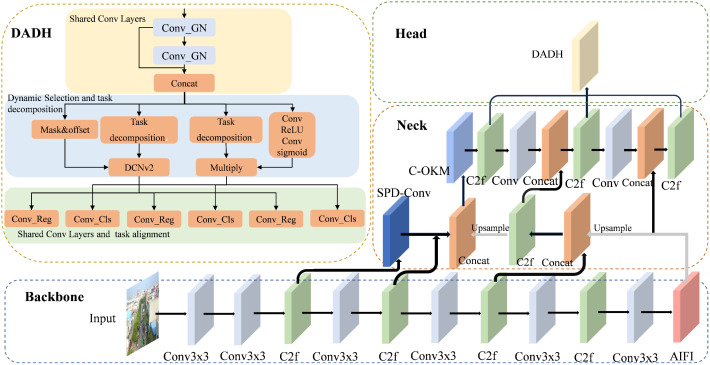
MFDA-YOLO network structure.

In this study, we propose the MFDA-YOLO model for UAV object detection based on YOLOv8. This model effectively solves two significant problems in UAV scenarios: the loss of small target features and computational constraints on edge devices. The overall network architecture of MFDA-YOLO is illustrated in Fig 2, with its core improvements permeating the backbone network, neck network, and detection head of the model. In the backbone network, we introduce the AIFI module, whose global attention mechanism enhances deep feature representations, effectively mitigating information loss in small targets caused by successive downsampling. Subsequently, the backbone-enhanced features are fed into the neck network, where they undergo processing by our specially designed DIDP module for small targets. This module efficiently restores and refines features, ensuring that minute details of small targets are preserved and effectively conveyed. Ultimately, these multi-scale refined features are input into the DADH detection head. By learning task-interactive features and employing a dynamic feature selection mechanism, this module significantly improves both classification and localization accuracy. Furthermore, the entire architecture is optimized using the WIoUv3 loss function, guiding the model to focus on challenging, complex targets during training and thereby further enhancing overall performance.

### 3.1 AIFI module

The high flight altitude of unmanned aerial vehicles renders targets minuscule, whilst the platform’s rapid movement obscures the fine textural details essential for identification. Though efficient, conventional SPPF modules frequently prove ineffective in such scenarios. Their repetitive pooling operations, designed for general feature extraction, may inadvertently erase the minute yet critical information required to define small aerial targets.

**Fig 3:**
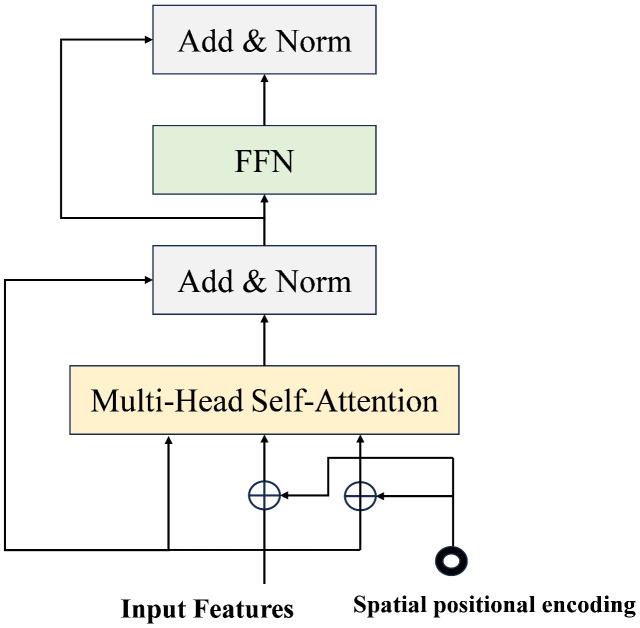
Structural diagram of the AIFI module.

To address this, we replace the traditional SPPF module with the AIFI module [18], which processes high-level semantic features through self-attention to effectively capture texture details in UAV detection. At the same time, to enable the AIFI module to extract key information more efficiently, we added a 1 × 1 convolution layer to the input to achieve channel compression. This achieves channel compression, filters out redundant information, and ensures the module can efficiently focus on the most salient features for drone detection. The AIFI structure is shown in Fig 3.

The AIFI model transforms the input 2D feature map $X\in R^{H\times W\times C}$ into a 1D feature sequence $X_{seq}\in R^{N\times C}$. Subsequently, the sequence is processed through a multi-head self-attention mechanism to learn positional correlations and generate attentional features. Residual concatenation and layer normalization are then performed to preserve the original feature information [19]. The feedforward network further introduces nonlinear transformations to learn complex correlations between feature sequences. Ultimately, the resulting sequence is reconstructed into a 2D feature map for effective fusion of global contextual information and local spatial structure. The mathematical representation of the AIFI module’s process is presented as follows:


$$Q,K,V=Flatten\cdot W^{Q},Flatten\cdot W^{K},Flatten\cdot W^{V}$$ (1)

Output=Re\nolimitsshape(MultiHeadatt(Q,K,V)) (2)

where ${\text{W}}^{Q},{\text{W}}^{K},{\text{W}}^{V}$ are linear transformation matrices. The Flatten operation reconstructs the multidimensional feature tensor into a one-dimensional vector through dimensionality reduction mapping. In contrast, the Reshape operation restructures the one-dimensional feature sequence into a spatial tensor whose dimensions match the structure of the original input vectors.

The AIFI module reduces the complexity of the model and improves the deep feature representation capability by increasing the internal scale interactions of the higher feature layers.

### 3.2 Drone image detection pyramid

While the AIFI module strengthens backbone features, effectively fusing them for small object detection remains a key challenge. Standard feature pyramids (P3-P5) lack the necessary resolution for small objects common in drone imagery. However, directly incorporating high-resolution P2 layers incurs prohibitive computational overhead, rendering it impractical for resource-constrained unmanned aerial vehicle platforms requiring real-time responsiveness.

To overcome these problems, we design the DIDP module for detecting small targets in UAV images. On the P2 detection layer, we apply SPD-Conv [20] to perform feature extraction and fuse it with the P3 detection layer. Meanwhile, in order to avoid feature degradation, we propose the C-OKM module. This module performs channel separation through a cross-stage partial network [21] and integrates the multi-scale perception capability of Omni-Kernel [22] to achieve efficient feature recovery.

#### 3.2.1 SPD-Conv module.

**Fig 4:**
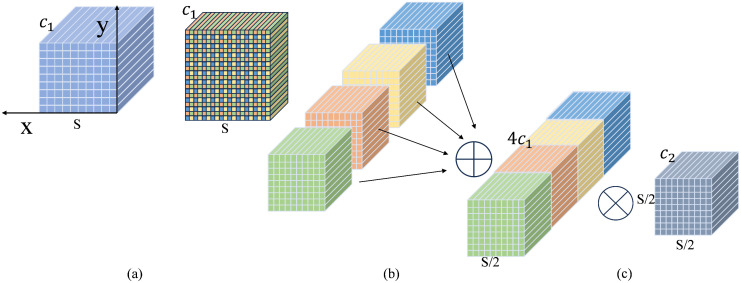
The SPD-Conv specific process when scale =  2.

The SPD-Conv extracts multi-scale features through spatial reorganization and convolution operations, which improves the detection accuracy of small targets in low-resolution images. The module comprises two core components: an SPD layer and a non-strided convolution (N-S Conv) layer. The workflow of SPD-Conv is illustrated in Fig 4.

The SPD layer decomposes the input feature map X of dimensions ${S\times S\times C}_{\text{1}}$ into multiple sub-feature maps $f_{x,y}$ as:


$$\left\{ \begin{array}{c} \begin{array}{c} {}*20c\;\;\;f_{0,0}=X\left[0:s:scale,0:S:scale\right],\ldots , \end{array}\\ f_{scale-1,0}=X\left[scale-1:S:scale,0:S:scale\right]\\ f_{0,1}=X\left[0:S:scale,1:S:scale\right],\ldots ,\\ f_{scale-1,1}=X\left[scale-1:S:scale,1:S:scale\right]\\ .\\ .\\ .\\ f_{0,scale-1}=X\left[0:S:scale,scale-1:S:scale\right],\ldots ,\\ f_{scale-1,scale-1}=X\left[scale-1:S:scale,scale-1:S:scale\right] \end{array}\right.$$ (3)

where scale is the down-sampling factor. Each sub-feature map $f_{x,y}$ consists of the original feature map elements X(i,j) satisfying that i+x and i+y are divisible by scale. The spatial dimension of a sub-feature map $f_{x,y}$ is $\frac{S}{scale}\times \frac{S}{scale}\times C_{\text{1}}$.

As shown in Fig 4(a), when scale = 2, the original feature map *X* is partitioned into four sub-feature maps $f_{\text{0},\text{0}}$, $f_{\text{1},\text{0}}$, $f_{\text{0},\text{1}}$, and $f_{\text{1},\text{1}}$, each with a dimension of $\frac{S}{\text{2}}\times \frac{S}{\text{2}}\times C_{\text{1}}$. Subsequently, the new feature map $X^{\prime }$ of size $\frac{S}{scale}\times \frac{S}{scale}\times scale^{2}C_{1}$ is generated by concatenating these sub-feature maps along channels as shown in Fig 4(b).

Next, the generated feature map $X^{\prime }$ is fed into an N-S Conv with $C_{\text{2}}$ filters. After the N-S Conv, the output feature map $X^{\prime \prime }$ has a size of $\frac{S}{scale}\times \frac{S}{scale}\times C_{\text{2}}$, as shown in Fig 4(c). This convolutional layer maximizes the retention of discriminative information in the input feature maps, and avoids the loss of small target features that can occur with standard hierarchical convolution.

#### 3.2.2 C-OKM module.

**Fig 5:**
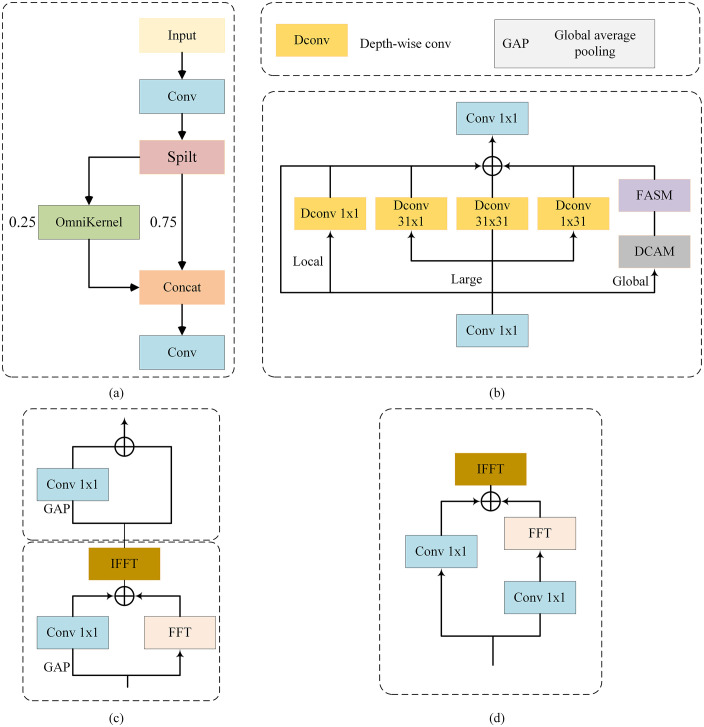
Details of the C-OKM module. **(a)**: C-OKM. **(b)**: Omni-Kernel module. **(c)**: DCAM. **(d)**: FASM.

However, following feature extraction and fusion, the features remain susceptible to degradation due to motion blur and jitter. To address this, we have designed the C-OKM module to perform image restoration. As shown in Fig 5, the C-OKM module adopts a multi-branch architecture, which can recover small target features to a great extent and maintain computational efficiency.

As illustrated in Fig 5(a), the cross-stage partial structure divides the input feature map into four channel slices. One of the slices is augmented by the Omni-Kernel module and fused with the other slices, to preserve the original features of the channel dimension. The Omni-Kernel module is shown in Fig 5(b). The input features are first transformed by a 1 × 1 convolutional layer and subsequently divided into three branches to capture local, large-scale, and global features separately. The outputs of each branch are fused by addition and further refined by another 1 × 1 convolutional layer.

In the local branch, we use a 1 × 1 Depthwise Separable Convolution (D-Conv) to enhance local image features. In the large branch, we employ a low-complexity larger odd-sized K × K D-Conv to capture large-scale features and expand the receptive field. Meanwhile, to efficiently capture contextual information and manage the computational overhead, we use 1 × 31 and 31 × 1 D-Conv in parallel at the bottleneck location.

In the global branch, the network is trained mainly on cropped image segments. During inference, input images are significantly larger in size than those used in training. This size discrepancy prevents the convolutional kernel from covering the entire global domain. Therefore, we introduce a dual-domain processing technique to enhance global modeling. Specifically, the global branch integrates two key modules: the dual-domain channel attention module (DCAM) in Fig 5(c) and the frequency-based spatial attention module (FSAM) in Fig 5(d).

The DCAM module first converts features to the frequency domain using the Fourier transform. It then reweights the frequency domain features using channel weights generated by global average pooling in the spatial domain. After that, secondary channel optimization is performed in the spatial domain. The FSAM module extracts global context in the frequency domain through dual paths and generates spatial domain importance masks. These masks are fused in the frequency domain and returned to the spatial domain after inverse transform.

### 3.3 Dynamic alignment detection head

The dynamic observation perspective of unmanned aerial vehicles exacerbates the inherent conflict between the classification and localization tasks within detection models. Drastic changes in target appearance amplify a core conflict: features cannot be both general enough for classification and precise enough for localization, which degrades localization accuracy.

**Fig 6:**
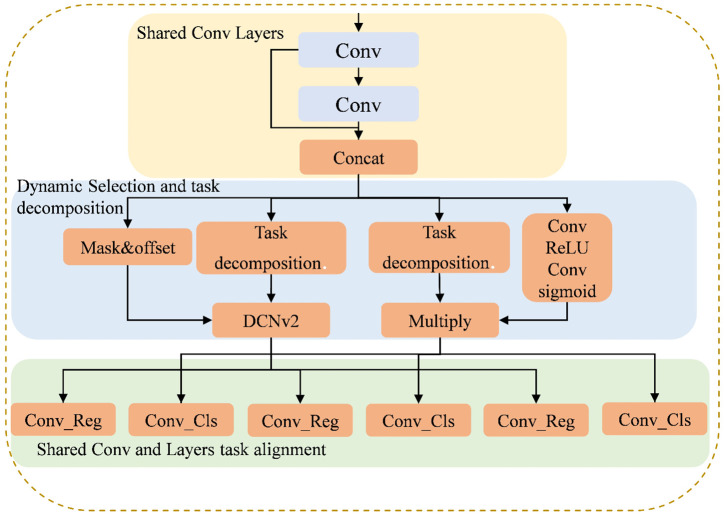
The structure of the DADH.

To solve this problem, we propose the DADH module by combining TOOD’s [23] interactive label assignment mechanism with task consistency optimization. Unlike dynamic heads relying on attention weighting (e.g., DyHead [9]), DADH integrates Deformable Convolutional Network v2 (DCNv2) [24] alongside task decomposition to dynamically optimize feature sampling for localization. The specific details of the DADH module are illustrated in Fig 6. First, multi-scale features are efficiently extracted through shared convolutional layers; subsequently, these features are fed into a task decomposition module, decoupling into two parallel branches for localization and classification. In the localization branch, we incorporate a DCNv2 to dynamically optimize the feature sampling region, thereby accommodating the complex geometric deformations of targets within aerial drone imagery. Concurrently, the classification branch generates more discriminative task-specific representations by dynamically weighting the shared features. Ultimately, the dynamic alignment process enhances feature consistency between the two parallel branches, enabling each to generate more precise classification and localization predictions.

#### 3.3.1 Shared convolutional layer.

In order to reduce the number of model parameters and to efficiently integrate multi-scale features, we design the shared convolutional layer. The input feature map undergoes a shared convolution for initial feature extraction, followed by group normalization [25] to separate channels into groups for intra-group standardization. After that, the processed feature maps perform convolution and group normalization operations again to further refine and extract deeper feature information. Finally, the refined features are concatenated with the original inputs along the channel dimension, to integrate hierarchical features and enhance representation capacity. The output feature map Y is computed by sliding the shared convolution kernel K over a local region of the input X, and can be expressed as:


$$Y_{i,j}={\left(K\times X\right)}_{i,j}$$ (4)

where (i,j) is the position on the output feature map Y. The final augmented feature map Y will serve as a unified input, fed into the subsequent dynamic selection and task decomposition module.

#### 3.3.2 Task decomposition.

**Fig 7:**
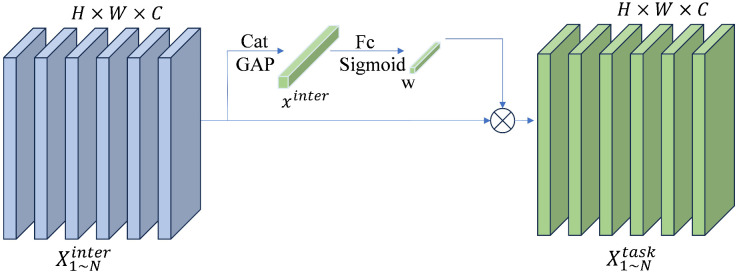
The principle of the task decomposition.

In single-branch networks, the divergent feature requirements of classification and localization tasks can lead to feature conflicts when they share the same set of features. To address this issue, we introduce the task decomposition whose core lies in introducing a layer-wise attention mechanism. This dynamically decouples shared task interaction features, thereby generating task-specific feature representations. The principle of the task decomposition is illustrated in Fig 7.

The task decomposition employs a layer-wise attention mechanism to compute separate task-specific features for classification and localization, thereby mitigating feature conflicts, as follows:


$$X_{task}=w_{k}X_{inter}^{k},k=1\ldots N$$ (5)

where $w_{k}$ denotes the kth element of the learning layer’s attention weight. $X_{inter}^{k}$ is the kth cross-layer feature. $X_{task}^{k}$ is the kth task-related feature. ω is the weight calculated as:


$$\omega =\sigma \left(fc_{2}\left(\delta \left(fc_{1}\left(x_{inter}\right)\right)\right)\right)$$ (6)

where $f_{C_{\text{1}}}$ and $f_{C_{\text{2}}}$ denote the two fully connected layers, σ represents the sigmoid function, δ denotes the non-linear factor, and $x_{inter}$ denotes the cascading features obtained from $X_{inter}$ by average pooling.

The classification and localization results are predicted based on${\;X}_{task}$,respectively:


$$Z_{task}=conv_{2}\left(\delta \left(conv_{1}\left(X_{task}\right)\right)\right)$$ (7)

where $X_{task}$ denotes the task-related features obtained by $X_{task}^{k}$ concatenation. $Conv_{\text{1}}$ is the 1 × 1 convolutional layer designed to reduce dimensionality, and $Conv_{\text{2}}$ is used for further feature transformation.

#### 3.3.3 Dynamic selection and task alignment.

Although task decomposition successfully provides distinct characteristics for different tasks, these features remain static in their processing approach. When confronted with dynamic scenarios during drone flight, where target posture and scale undergo abrupt changes, fixed receptive fields struggle to accurately capture rapidly deforming or moving targets. To address these issues, we introduce the DCNv2 to dynamically adjust the interaction characteristics in the localization branch after the task decomposition. DCNv2 leverages interaction features learned from the feature extractor to generate offsets and masks, which enables efficient dynamic feature selection, which can be expressed as:


$$y\left(p\right)=\sum_{k=1}^{K}w_{k}\cdot x\left(p+p_{k}+\Delta p_{k}\right)\cdot \Delta m_{k}$$ (8)

where x and y represent the input and output feature maps respectively, p denotes the position on the feature map, k represents the convolution kernel size, $w_{k}$ is the weight of the convolution kernel, $p_{k}$ denotes the predefined offset at the kth position, $\Delta p_{k}$ is the offset for adjusting the sampling position, and $\Delta m_{k}$ is the mask for dynamically adjusting the feature weights.

In the classification branch, the interaction features learned from the shared convolutional layer are dynamically selected and integrated with the decomposed task-specific features. First, a 1 × 1 convolution reduces the channel dimension of high-level features to one-fourth of the original. The compressed features are then activated by ReLU and processed by a 3 × 3 convolution to integrate spatial context. Finally, a Sigmoid function normalizes the output to generate a pixel-wise category attention mask in the (0,1) range. In the feature fusion stage, element-wise multiplication is performed between this mask and the main branch features to achieve dynamic weighting.

DADH achieves task decomposition by dynamically computing specific features for different tasks, enabling the feature extraction process to be adjusted according to the requirements of each specific task. It reduces interference between task features and improves execution efficiency.

### 3.4 WIoUv3 loss

The drastic scale variations and densely overlapping targets in drone imagery pose significant challenges for bounding box regression. The default CIoU loss function of YOLOv8 is particularly susceptible to these issues, tending to converge to local optima in crowded scenes, resulting in suboptimal localization accuracy.

To address these limitations, we introduce WIoUv3 [26], a loss function using a dynamic non-monotonic focusing strategy. This design enhances the model’s adaptability by focusing on sample quality and mitigating the excessive gradients often assigned to low-quality samples.

The WIoUv3 loss function evaluates the quality of candidate anchor boxes through outlierness degrees. A lower outlierness degree corresponds to a higher-quality anchor box, whereas a higher outlierness degree reflects lower-quality [27]. The definition of the outlierness degree β is shown as:


$$\beta =\frac{L_{IoU}^{*}}{\overset{―}{L_{IoU}}}$$ (9)

where $\;L_{IoU}^{*}$ indicates the current *IoU* loss value. The normalization factor $\overset{―}{L_{IoU}}$ is the exponential moving average of the $L_{IoU}$.

This outlierness metric mechanism implements an intelligent gradient allocation strategy. Specifically, it allocates higher gradient gains to anchor boxes with moderate β values, as these samples hold the greatest value for model optimization. Conversely, the mechanism suppresses gradients from well-matched (high-quality) and difficult-to-correct (low-quality) anchor boxes. This strategy aims to eliminate misleading gradients arising from target overlap or occlusion in crowded scenes, which are key contributors to positioning inaccuracies [28]. By focusing learning effort on informative yet learnable samples, the model avoids over-optimizing either easy samples or intractable outliers. The non-monotonic focusing factor r is defined as follows:


$$r=\frac{\beta }{\delta \alpha^{\beta -\delta }}$$ (10)

where α and δ represent hyperparameters. The α is used to adjust the gradient gain amplitude corresponding to objects of different sizes. And δ governs the curvature of the gradient response function to concentrate optimization focus within targeted IoU intervals.

The WIoUv3, which utilizes a geometric penalty based on the distance metric along with a non-monotonic focusing factor r, is defined as follows:


$$R_{WIoU}=exp\left(\frac{{\left(x-x_{gt}\right)}^{2}+{\left(y-y_{gt}\right)}^{2}}{{\left(W_{g}^{2}+H_{g}^{2}\right)}^{*}}\right)$$ (11)

$$L_{WIoU}^{v3}=rR_{WIoU}L_{IoU}$$ (12)

where (*x*, *y*) and $(x_{gt}$,$\;y_{gt}$) denote the predicted and ground truth bounding box center coordinates, respectively. $W_{g}$ and $H_{g}$ are the minimum bounding box width and height. The asterisk (*) indicates a separation operation on the gradient. $R_{WIoU}$ is the attention factor, which measures the distance between the predicted bounding boxes and the ground truth bounding boxes.

By leveraging the dynamic characteristics of IoU and the optimization criterion of anchor boxes, WIoUv3 dynamically allocates gradients during training, which improves UAV object detection performance.

## 4. Experiment

### 4.1 Experimental configuration

The experimental platform is equipped with an Intel Core i9-13900K processor, 32 GB RAM, and an NVIDIA GeForce RTX 4090 to provide powerful computing support. All input images are standardized to 640 × 640, with a batch size of 32 and 500 training epochs. The optimizer employs stochastic gradient descent with an initial learning rate of 0.01, momentum of 0.937, and weight decay of 0.0005. Additionally, the IOU threshold was set to 0.7, while the hyperparameters α and δ of the WIOUv3 loss function were configured to 1.7 and 2.7, respectively. The software environment is Python 3.10.14 and PyTorch with CUDA 12.1. These experimental conditions laid a solid foundation for subsequent comparison experiments.

### 4.2 Dataset

To comprehensively evaluate the performance of the proposed UAV object detection model, we conduct experimental validation on three datasets, Visdrone2019, HIT-UAV and NWPU VHR-10.

The Visdrone2019 [29] dataset is widely used in UAV object detection research. Visdrone2019 contains 8,599 images covering a wide range of UAV scenes (urban, outdoor, indoor, factory, laboratory, etc.), weather conditions (daytime, nighttime, sunny, cloudy, rainy, etc.), as well as different light intensities and shooting angles. The dataset contains 6,471 training images, 548 validation images, and 1,610 test images. The annotations include 10 target categories: pedestrians, people, bicycles, cars, vans, trucks, tricycles, awning tricycles, buses and motors.

The HIT-UAV [30] dataset consists of 2898 infrared thermograms acquired by UAVs. It significantly broadens the UAV scenarios in low-light environments. The HIT-UAV dataset contains numerous small objects, which roughly include five main categories: humans, vehicles, bicycles, other vehicles, and dontcare. The dataset is divided into 2029 training images, 290 validation images, and 579 test images.

NWPU VHR-10 [31] is a high-resolution remote sensing dataset, comprising 650 annotated images and 150 unlabeled images. These images were extracted from the Google Earth and Vaihingen datasets, encompassing a total of 3,651 instances. NWPU VHR-10 covers ten different categories, such as tennis courts, airplanes, ships, basketball courts, and athletics tracks.

### 4.3 Evaluation indicators

To assess the performance of the MFDA model, we use Precision (P), Recall (R), mean Average Precision (mAP), and its variants mAP0.5 and mAP0.5:0.95 as evaluation metrics [32].

P represents the ratio of true positive samples to all predicted positive samples, computed as follows:


$$Precision=\frac{TP}{TP+FP}$$ (13)

where TP represents the number of correctly predicted positive samples, and FP denotes the number of negative samples that are erroneously classified as positive.

R is the ratio of the number of correctly identified positive samples to the total number of actual positive samples, expressed as follows:


$$Recall=\frac{TP}{TP+FN}$$ (14)

where FN denotes the number of positive samples incorrectly predicted as negative.

The mAP is the mean of the Average Precision (AP) across all categories, defined as follows:


$$mAP=\frac{\text{1}}{N}\sum_{i=1}^{N}AP_{i}$$ (15)

where i stands for the category index, and N represents the total category count in the training set.

The mAP0.5 measures the average accuracy when the IoU threshold is set at 0.5, and mAP0.5:0.95 assesses the average accuracy across IoU thresholds from 0.5 to 0.95.

### 4.4 Experimental results and analysis

#### 4.4.1 Ablation experiments.

We evaluated hyperparameters α and δ in WIoUv3 to assess their impact on detection accuracy. Experiments tested key combinations on the VisDrone2019 dataset.

As shown in Table 1, the parameter combination α=1.7, δ=2.7 achieved the best overall performance in all test combinations. Therefore, we adopted this parameter setting in all subsequent experiments and final models in this paper.

**Table 1:** Ablation study on hyperparameters of WIoUv3.

Hyperparameter	P	R	mAP0.5	mAP0.5:0.95
α=1.9,δ =3.0	0.435	0.329	0.310	0.178
α=1.6,δ =4.0	0.431	0.333	0.312	**0.180**
α=2.5,δ =2.0	0.426	0.334	0.311	0.179
α=1.7,δ =2.7	**0.438**	**0.338**	**0.317**	**0.180**

**Bold numbers: Best performance.**

To verify the effectiveness of the proposed AIFI, DIDP, DADH, and WIoUv3 modules on the MFDA-YOLO model, the following ablation experiments are performed on the VisDrone2019 dataset. The detailed experimental results are summarized in Table 2.

**Table 2:** Ablation experiment results of modules on the VisDrone2019-DET-Test.

Models	AIFI	DIDP	DADH	WIoUv3	P	R	mAP0.5	mAP0.5:0.95	Params/M
Yolov8n					0.393	0.296	0.273	0.153	3.01
✓	✓				0.392	0.292	0.270	0.152	2.93
✓		✓			0.431	0.322	0.303	0.172	3.31
✓			✓		0.398	0.303	0.279	0.158	2.24
✓				✓	0.411	0.291	0.276	0.152	3.01
✓		✓	✓		0.434	0.328	0.308	0.177	2.54
✓	✓		✓		0.403	0.303	0.283	0.160	2.19
✓	✓			✓	0.398	0.300	0.279	0.155	2.93
✓		✓		✓	0.428	0.319	0.301	0.170	3.31
✓		✓	✓	✓	0.427	0.330	0.307	0.174	2.54
✓	✓	✓	✓		0.433	0.334	0.310	0.177	2.49
✓	✓	✓	✓	✓	**0.438**	**0.338**	**0.317**	**0.180**	**2.49**

Table 2 shows that the AIFI structure effectively reduces the number of model parameters. The DIDP module increases the mAP0.5 by 3 percentage points, which demonstrates its advantages for small-target feature extraction. The DADH module reduces the number of parameters by 25.6% compared to the baseline model, which thereby meets the requirements for lightweight UAV detection. In addition, the WIoUv3 loss function effectively improves mAP0.5 by 0.3 percentage points compared to the baseline, which allows the model to better focus on small targets. The experimental results show that the mAP0.5 of MFDA-YOLO is 4.4 percentage points higher than that of YOLOv8n. Furthermore, R and P reach an optimum level with a 17.2% reduction in the number of parameters.

#### 4.4.2 Comparison experiments.

To evaluate the effectiveness of the proposed method, extensive comparative experiments are conducted. These comparison methods include several versions of the YOLO family, such as YOLOv5s, YOLOv5n, YOLOv8n [14], YOLOv9-t [33], YOLOv10n [34], YOLOX [35], YOLOv11n [36], YOLOv12n [37], and YOLOv13n [38], as well as other models like FCOS [7] and Retina-Net [39]. The performance of each model is comprehensively evaluated focusing on params, precision, FPS, mAP0.5, and mAP0.5:0.95, and the results are presented in Table 3 under the Visdrone2019-DET-Test dataset.

**Table 3:** Results of different models on the VisDrone2019-DET-Test.

Models	Params/M	R	P	FPS	mAP0.5	mAP0.5:0.95
Retina-Net	36.51	0.306	0.379	59	0.281	0.161
FCOS	32.13	0.331	0.409	60	0.309	0.174
YOLOv5n	**1.77**	0.263	0.349	227	0.233	0.118
YOLOv5s	7.03	0.320	0.410	222	0.291	0.156
YOLOX	5.03	0.316	0.434	181	0.302	0.163
YOLOv8n	3.01	0.296	0.393	**277**	0.273	0.153
YOLOv9-t	2.62	0.308	0.427	106	0.294	0.170
YOLOv10n	2.69	0.297	0.399	256	0.273	0.153
YOLOv11n	2.58	0.301	0.399	217	0.276	0.153
YOLOv12n	2.51	0.293	0.387	208	0.268	0.152
YOLOv13n	2.45	0.285	0.383	156	0.261	0.145
MFDA-YOLO	2.49	**0.338**	**0.438**	149	**0.317**	**0.180**

The experiment results show that Retina-Net and FCOS are not suitable for real-time UAV object detection due to more parameters. MFDA-YOLO achieves a better balance between parameters and detection accuracy, with only 2.49M parameters while attaining the mAP0.5 of 0.317 and the mAP0.5:0.95 of 0.180. This performance outperforms recent YOLO variants such as YOLOv12n and YOLOv13n. Meanwhile, its lightweight design enhances small-target detection in UAV scenarios, which achieves real-time performance with 149 FPS and improves precision by 4.5 percentage points.

**Fig 8:**
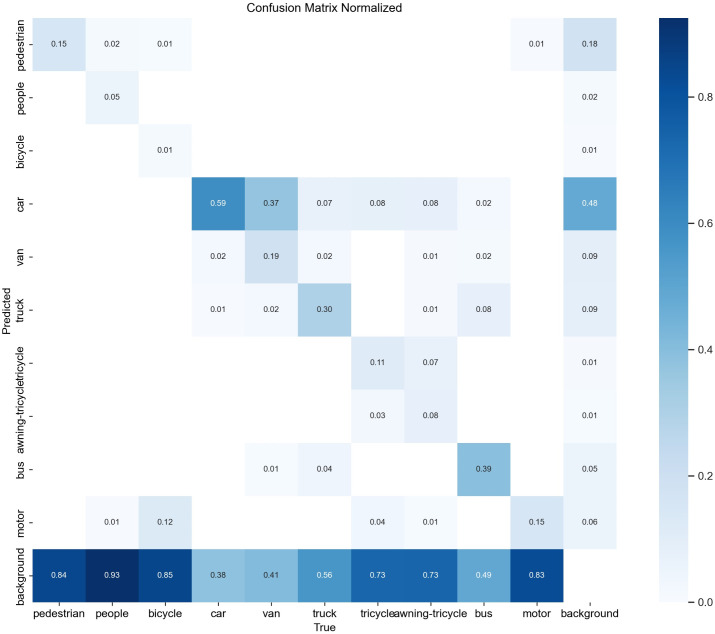
Confusion matrix of YOLOv8n.

**Fig 9:**
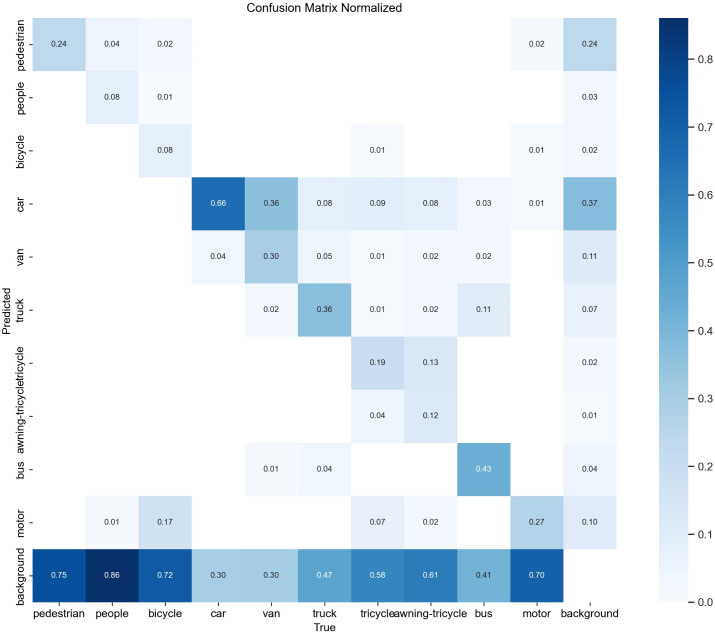
Confusion matrix of MFDA-YOLO.

In order to visualize the effectiveness of the MFDA-YOLO model in solving the leakage and misdetection problems, we compared it with the YOLOv8n on the confusion matrix. The results are shown in Fig 8 and Fig 9.

The MFDA-YOLO significantly improves the classification accuracy and reduces the inter-class confusion rate. The results show that the precision for “Pedestrian”, “Van”, and “Car” is increased by 9, 11, and 7 percentage points, respectively. The category “Car” has the highest classification accuracy of 0.66. In dense target scenarios, precision for “motorcycle” and “bicycle” is improved by 12 and 7 percentage points, respectively. In occluded environments, false detections of “Tricycle” are decreased by 13 percentage points. In summary, the MFDA-YOLO model effectively reduces the leakage and false detection in UAV object detection.

### 4.5 Generalization experiments

To fully validate the effectiveness and robustness of MFDA-YOLO, we conduct generalization experiments on the HIT-UAV [30] and NWPU VHR-10 [31] datasets. Specific performance results for each model on these datasets are shown in Table 4 and Table 5.

**Table 4:** Results of different models on the HIT-UAV.

Models	Params/M	P	R	mAP0.5	mAP0.5:0.95
RTMDet	4.84	0.866	0.767	0.823	0.528
YOLOv5n	**1.77**	0.851	0.784	0.820	0.514
YOLOv5s	7.02	0.882	**0.807**	0.834	0.545
YOLOX	5.03	0.831	0.692	0.752	0.435
YOLOv8n	3.01	0.854	0.805	0.825	0.548
YOLOv9-t	2.62	0.840	0.794	0.820	0.542
YOLOv10n	2.70	0.879	0.755	0.800	0.526
YOLOv11n	2.58	0.875	0.753	0.803	0.529
YOLOv12n	2.51	0.823	0.789	0.820	0.537
YOLOv13n	2.45	0.867	0.759	0.820	0.536
MFDA-YOLO	2.49	**0.901**	0.802	**0.863**	**0.570**

**Table 5:** Results of different models on the NWPU VHR-10.

Models	Params/M	P	R	mAP0.5	mAP0.5:0.95
DETR	41.56	0.885	**0.859**	0.882	0.549
ATSS	32.13	0.796	0.797	0.822	0.469
YOLOv5n	**1.77**	0.879	0.814	0.853	0.499
YOLOX	5.03	**0.909**	0.819	0.888	0.543
TOOD	32.03	0.803	0.791	0.827	0.474
YOLOv8n	3.01	0.904	0.808	0.880	0.563
YOLOv9-t	2.62	0.899	0.815	0.878	**0.565**
YOLOv10n	2.70	0.851	0.783	0.823	0.511
YOLOv11n	2.58	0.872	0.853	0.884	0.552
YOLOv12n	2.51	0.873	0.805	0.861	0.527
YOLOv13n	2.45	0.823	0.784	0.821	0.504
MFDA-YOLO	2.49	0.900	0.831	**0.889**	0.545

Compared with the YOLOv8 baseline, MFDA-YOLO achieves improvements of 3.8 percentage points in mAP0.5 and 2.2 percentage points in mAP0.5:0.95. The MFDA-YOLO model achieves the highest mAP0.5 of 0.863 and mAP0.5:0.95 of 0.570, which outperforms advanced models such as RTMDet [40], YOLOv9-t [33], YOLOv10n [34], YOLOv11n [36], YOLOv12n [37] and YOLOv13n [38]. This demonstrates MFDA-YOLO’s superior performance in infrared-based UAV object detection.

We conduct a comprehensive comparison with several state-of-the-art models for object detection in Table 5, which includes DETR [41], ATSS [42], YOLOv5n, YOLOX [35], TOOD [23], YOLOv8n [14], YOLOv9-t [33], YOLOv10n [34], YOLOv11n [36], YOLOv12n [37] and YOLOv13n [38].

As shown in Table 5, the DETR model achieves the R as high as 0.859, but its large number of parameters makes it difficult to deploy in real-world scenarios. The YOLOX model achieves a P of 0.909, but its R is relatively low. The YOLOv11n model achieves the mAP0.5 of 0.884, but its P is only 0.872. Compared with the baseline model YOLOv8n, the MFDA-YOLO model improves R by 2.3 percentage points and achieves the highest mAP0.5 of 0.889. The experimental results validate the broad applicability of MFDA-YOLO in remote sensing scenarios.

### 4.6 Visualization

**Fig 10:**
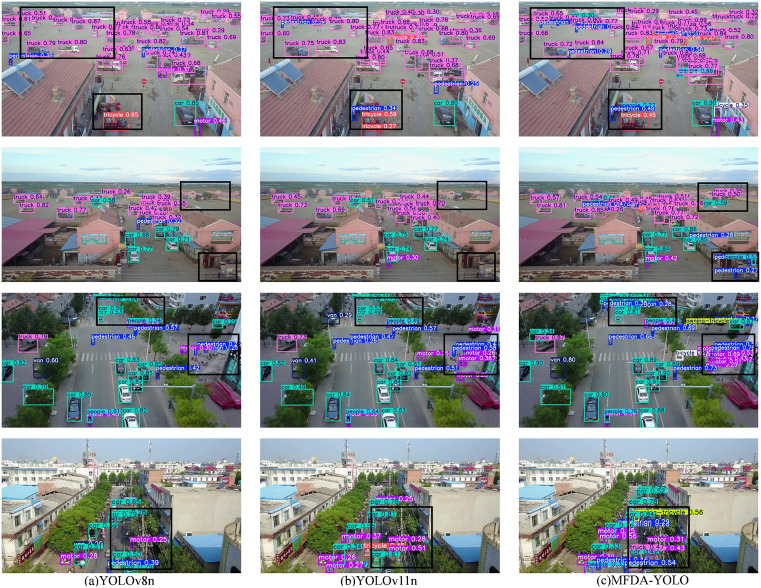
Comparison of detection results across different models on the Visdrone2019 dataset. (The black box demonstrates the MFDA-YOLO’s ability to reduce missed and false detections).

To thoroughly assess the reliability and flexibility of the object detection model in UAV scenarios, we conduct systematic multi-environment tests. Fig 10 presents the object detection capability of the MFDA-YOLO model in various challenging environments. Through detailed visualization analyses of detection results across different geographic locations and UAV flight altitudes, we find that the MFDA-YOLO model demonstrates high accuracy in detecting dense and small objects in complex environments.

The MFDA-YOLO model exhibits excellent detection performance in dense environments and is well-suited for applications in UAV object detection. In dense crowds and vehicle scenarios, we find that the MFDA-YOLO model effectively identifies small targets such as pedestrians and motorbikes categories, which are often missed by the YOLOv8n and YOLOv11n models. Additionally, it successfully reduces the misclassification of vehicles.

**Fig 11:**
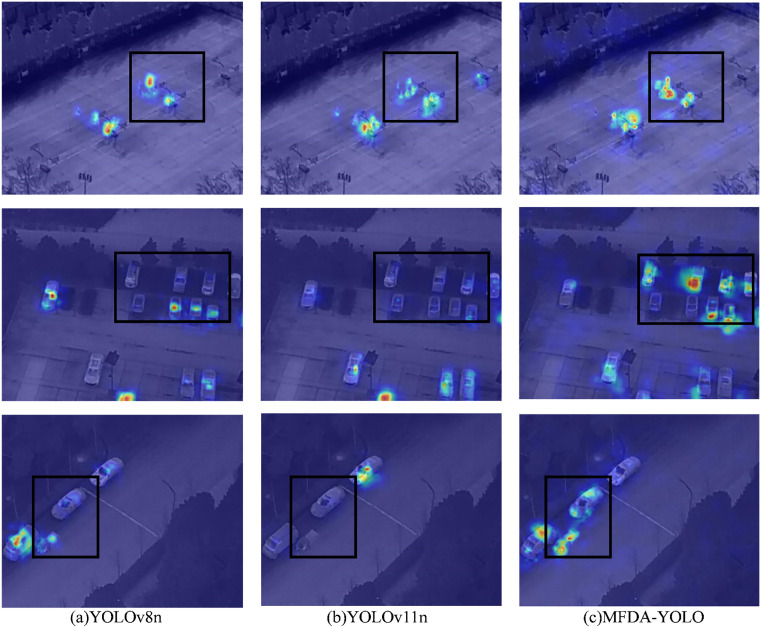
Heat map comparison among different models on the HIT-UAV dataset. (The black bounding box highlights that MFDA-YOLO produces markedly more concentrated heat-maps on small objects).

In order to verify the performance of the MFDA-YOLO model in the infrared environment, we perform a comprehensive thermogram analysis of YOLOv8n, YOLOv11n, and MFDA-YOLO, and the results are shown in Fig 11.

In the first row of images, the MFDA-YOLO model is able to detect more small targets. In the second row of images, YOLOv8n exhibits a significant lack of attention when handling dense scenarios, which results in a high leakage rate and false detections. In the third row of images, leakage detection is present in both YOLOv8n and YOLOv11n. In contrast, the MFDA-YOLO model detects most targets and reduces leakage and false detections. Overall, the MFDA-YOLO model can pay more attention to fine-grained details and have a broader detection scope, which shows better detection performance compared to YOLOv8n and YOLOv11n.

## 5. Conclusion

This study proposes an object detection model for UAV aerial scenes based on YOLOv8n. We incorporate the AIFI feature interaction module in the backbone network to enhance the feature representation capability. The DIDP module uses SPD-Conv to transfer small target features from the P2 layer to the P3 layer for feature fusion. It then uses the C-OKM module to recover the missing feature information. We design a DADH module, which learns task interaction features from shared convolutional layers and selects them dynamically to reduce model parameters. Additionally, we utilize the WIoUv3 loss function to improve the model’s performance for focusing on challenging small targets.

The MFDA-YOLO model demonstrates 4.4 percentage points and 2.7 percentage points improvements in mAP0.5 and mAP0.5:0.95 on VisDrone2019, and achieves the highest mAP0.5 on both HIT-UAV and NWPU VHR-10 datasets. The model reduces the parameters by 17.2% compared to the baseline, which ensures real-time performance.

Our future research focuses on dynamic adaptive mechanisms and model pruning techniques to build lightweight detection networks, which can achieve efficient deployment on low-computing platforms such as UAVs and edge devices.

## Supporting information

S1 File Experimental parameters. The specific parameters of the experiment and the configuration file.
(RAR)

## References

[1] Liu J, Zheng H. EFN: field-based object detection for aerial images. Remote Sensing. 2020;12(21):3630. doi: 10.3390/rs12213630

[2] Cheng G, Han J, Lu X. Remote sensing image scene classification: benchmark and state of the art. Proc IEEE. 2017;105(10):1865–83. doi: 10.1109/jproc.2017.2675998

[3] Ma S, Lu H, Liu J, Zhu Y, Sang P. LAYN: lightweight multi-scale attention YOLOv8 network for small object detection. IEEE Access. 2024;12:29294–307. doi: 10.1109/access.2024.3368848

[4] Doherty J, Gardiner B, Kerr E, Siddique N. BiFPN-YOLO: one-stage object detection integrating bi-directional feature pyramid networks. Pattern Recog. 2025;160:111209. doi: 10.1016/j.patcog.2024.111209

[5] Redmon J, Divvala S, Girshick R, Farhadi A. You only look once: unified, real-time object detection. In: 2016 IEEE conference on computer vision and pattern recognition (CVPR), 2016. 779–88. doi: 10.1109/cvpr.2016.91

[6] Law H, Deng J. CornerNet: detecting objects as paired keypoints. Int J Comput Vis. 2019;128(3):642–56. doi: 10.1007/s11263-019-01204-1

[7] Tian Z, Shen C, Chen H, He T. FCOS: fully convolutional one-stage object detection. In: 2019 IEEE/CVF International Conference on Computer Vision (ICCV), 2019. doi: 10.1109/iccv.2019.00972

[8] Tan M, Pang R, Le QV. EfficientDet: scalable and efficient object detection. In: 2020 IEEE/CVF Conference on Computer Vision and Pattern Recognition (CVPR), 2020. 10778–87. doi: 10.1109/cvpr42600.2020.01079

[9] Zhang Z, Zhu W. YOLO-MFD: remote sensing image object detection with multi-scale fusion dynamic head. Compt Mater Contin. 2024;79(2):2547–63. doi: 10.32604/cmc.2024.048755

[10] Cai Z, Vasconcelos N. Cascade R-CNN: high quality object detection and instance segmentation. IEEE Trans Pattern Anal Mach Intell. 2021;43(5):1483–98. doi: 10.1109/TPAMI.2019.2956516 31794388

[11] Lin T-Y, Dollar P, Girshick R, He K, Hariharan B, Belongie S. Feature pyramid networks for object detection. In: 2017 IEEE Conference on Computer Vision and Pattern Recognition (CVPR), 2017. 936–44. doi: 10.1109/cvpr.2017.106

[12] Liu Z, Lin Y, Cao Y, Hu H, Wei Y, Zhang Z, et al. Swin transformer: hierarchical vision transformer using shifted windows. In: 2021 IEEE/CVF International Conference on Computer Vision (ICCV), 2021. 9992–10002. doi: 10.1109/iccv48922.2021.00986

[13] Shi H, Yang W, Chen D, Wang M. ASG-YOLOv5: Improved YOLOv5 unmanned aerial vehicle remote sensing aerial images scenario for small object detection based on attention and spatial gating. PLoS One. 2024;19(6):e0298698. doi: 10.1371/journal.pone.0298698 38829850 PMC11146694

[14] Jocher G, Chaurasia A, Qiu J. YOLOv8: ultralytics official implementation. 2023. https://github.com/ultralytics/ultralytics

[15] Shamta I, Demir BE. Development of a deep learning-based surveillance system for forest fire detection and monitoring using UAV. PLoS One. 2024;19(3):e0299058. doi: 10.1371/journal.pone.0299058 38470887 PMC10931456

[16] Zhao X, Chen Y. YOLO-DroneMS: multi-scale object detection network for unmanned aerial vehicle (UAV) images. Drones. 2024;8(11):609. doi: 10.3390/drones8110609

[17] Zhang H, Sun W, Sun C, He R, Zhang Y. HSP-YOLOv8: UAV aerial photography small target detection algorithm. Drones. 2024;8(9):453. doi: 10.3390/drones8090453

[18] Zhao Y, Lv W, Xu S, Wei J, Wang G, Dang Q, et al. DETRs beat YOLOs on real-time object detection. In: 2024 IEEE/CVF Conference on Computer Vision and Pattern Recognition (CVPR), 2024. 16965–74. doi: 10.1109/cvpr52733.2024.01605

[19] Wang S, Jiang H, Li Z, Yang J, Ma X, Chen J, et al. PHSI-RTDETR: a lightweight infrared small target detection algorithm based on UAV aerial photography. Drones. 2024;8(6):240. doi: 10.3390/drones8060240

[20] Sunkara R, Luo T. No more strided convolutions or pooling: a new CNN building block for low-resolution images and small objects. In: Joint European Conference on Machine Learning and Knowledge Discovery in Databases. Springer; 2022.doi: 10.1007/978-3-031-26409-2_27

[21] Wang C-Y, Mark Liao H-Y, Wu Y-H, Chen P-Y, Hsieh J-W, Yeh I-H. CSPNet: a new backbone that can enhance learning capability of CNN. In: 2020 IEEE/CVF Conference on Computer Vision and Pattern Recognition Workshops (CVPRW), 2020. doi: 10.1109/cvprw50498.2020.00203

[22] Cui Y, Ren W, Knoll A. Omni-kernel network for image restoration. AAAI. 2024;38(2):1426–34. doi: 10.1609/aaai.v38i2.27907

[23] Feng C, Zhong Y, Gao Y, Scott MR, Huang W. TOOD: task-aligned one-stage object detection. In: 2021 IEEE/CVF International Conference on Computer Vision (ICCV), 2021. doi: 10.1109/iccv48922.2021.00349

[24] Zhu X, Hu H, Lin S, Dai J. Deformable ConvNets V2: more deformable, better results. In: 2019 IEEE/CVF Conference on Computer Vision and Pattern Recognition (CVPR), 2019. 9300–8. doi: 10.1109/cvpr.2019.00953

[25] Wu Y, He K. Group normalization. Lecture Notes in Computer Science. Springer International Publishing; 2018. 3–19. doi: 10.1007/978-3-030-01261-8_1

[26] Tong Z, Chen Y, Xu Z, Yu RJ. Wise-IoU: bounding box regression loss with dynamic focusing mechanism. arXiv preprint. 2023. doi: 10.48550/arXiv.2301.10051

[27] Shi M, Zheng D, Wu T, Zhang W, Fu R, Huang K. Small object detection algorithm incorporating swin transformer for tea buds. PLoS One. 2024;19(3):e0299902. doi: 10.1371/journal.pone.0299902 38512917 PMC10956868

[28] Liu C, Meng F, Zhu Z, Zhou L. Object detection of UAV aerial image based on YOLOv8. Front Compt Intell Syst. 2023;5(3):46–50. doi: 10.54097/fcis.v5i3.13852

[29] Du D, Zhu P, Wen L, Bian X, Lin H, Hu Q, et al. VisDrone-DET2019: the vision meets drone object detection in image challenge results. In: 2019 IEEE/CVF International Conference on Computer Vision Workshop (ICCVW), 2019. doi: 10.1109/iccvw.2019.00030

[30] Suo J, Wang T, Zhang X, Chen H, Zhou W, Shi W. HIT-UAV: a high-altitude infrared thermal dataset for unmanned aerial vehicle-based object detection. Sci Data. 2023;10(1):227. doi: 10.1038/s41597-023-02066-6 37080987 PMC10119175

[31] Cheng G, Han J, Zhou P, Guo L. Multi-class geospatial object detection and geographic image classification based on collection of part detectors. ISPRS J Photogram Remote Sens. 2014;98:119–32. doi: 10.1016/j.isprsjprs.2014.10.002

[32] Yacouby R, Axman D. Probabilistic extension of precision, recall, and f1 score for more thorough evaluation of classification models. In: Proceedings of the First Workshop on Evaluation and Comparison of NLP Systems, 2020. doi: 10.18653/v1/2020.eval4nlp-1.9

[33] Wang CY, Yeh IH, Liao HY. YOLOv9: learning what you want to learn using programmable gradient information. arXiv preprint. 2024. doi: arXiv:2402.13616

[34] Wang A, Chen H, Liu L, Chen K, Lin Z, Han J. YOLOv10: real-time end-to-end object detection. arXiv preprint. 2024. doi: 10.48550/arXiv.2405.14458

[35] Ge Z, Liu S, Wang F, Li Z, Sun J. YOLOX: Exceeding YOLO series in 2021. arXiv preprint. 2021. doi: 10.48550/arXiv.2107.08430

[36] Khanam R, Hussain M. YOLOv11: an overview of the key architectural enhancements. arXiv preprint. 2024. doi: 10.48550/arXiv.2410.17725

[37] Tian Y, Ye Q, Doermann D. YOLOv12: Attention-centric real-time object detectors. arXiv preprint. 2025. doi: 10.48550/arXiv.2502.12524

[38] Lei M, Li S, Wu Y, Hu H, Zhou Y, Zheng X. YOLOv13: real-time object detection with hypergraph-enhanced adaptive visual perception. arXiv preprint. 2025. doi: 10.48550/arXiv.2506.17733

[39] Lin T-Y, Goyal P, Girshick R, He K, Dollar P. Focal loss for dense object detection. In: 2017 IEEE International Conference on Computer Vision (ICCV), 2017. 2999–3007. doi: 10.1109/iccv.2017.324

[40] Lyu C, Zhang W, Huang H, Zhou Y, Wang Y, Liu Y. Rtmdet: an empirical study of designing real-time object detectors. arXiv preprint. 2022. doi: 10.48550/arXiv.2212.07784

[41] Zhu X, Su W, Lu L, Li B, Wang X, Dai J, et al. Deformable detr: deformable transformers for end-to-end object detection. arXiv preprint. 2020. doi: 10.48550/arXiv.2010.04159

[42] Zhang S, Chi C, Yao Y, Lei Z, Li SZ. Bridging the gap between anchor-based and anchor-free detection via adaptive training sample selection. In: 2020 IEEE/CVF Conference on Computer Vision and Pattern Recognition (CVPR), 2020. doi: 10.1109/cvpr42600.2020.00978

